# The ubiquitin ligase VviPUB19 negatively regulates grape cold tolerance by affecting the stability of ICEs and CBFs

**DOI:** 10.1093/hr/uhae297

**Published:** 2024-10-23

**Authors:** Ling Wang, Mengyu Zhao, Xue Zhang, Ting Zhao, Congbo Huang, Yujin Tang, Yan Li, Chaohong Zhang

**Affiliations:** State Key Laboratory of Crop Stress Resistance and High-Efficiency Production, College of Horticulture, Northwest A&F University, Yangling, Shaanxi, China; Key Laboratory of Biology and Genetic Improvement of Horticultural Crops (Northwest Region), Ministry of Agriculture, Yangling, Shaanxi, China; College of Horticulture and Landscape Architecture, Henan Institute of Science and Technology, Xinxiang, China; State Key Laboratory of Crop Stress Resistance and High-Efficiency Production, College of Horticulture, Northwest A&F University, Yangling, Shaanxi, China; Key Laboratory of Biology and Genetic Improvement of Horticultural Crops (Northwest Region), Ministry of Agriculture, Yangling, Shaanxi, China; State Key Laboratory of Crop Stress Resistance and High-Efficiency Production, College of Horticulture, Northwest A&F University, Yangling, Shaanxi, China; Key Laboratory of Biology and Genetic Improvement of Horticultural Crops (Northwest Region), Ministry of Agriculture, Yangling, Shaanxi, China; State Key Laboratory of Crop Stress Resistance and High-Efficiency Production, College of Horticulture, Northwest A&F University, Yangling, Shaanxi, China; Key Laboratory of Biology and Genetic Improvement of Horticultural Crops (Northwest Region), Ministry of Agriculture, Yangling, Shaanxi, China; State Key Laboratory of Crop Stress Resistance and High-Efficiency Production, College of Horticulture, Northwest A&F University, Yangling, Shaanxi, China; Key Laboratory of Biology and Genetic Improvement of Horticultural Crops (Northwest Region), Ministry of Agriculture, Yangling, Shaanxi, China; State Key Laboratory of Crop Stress Resistance and High-Efficiency Production, College of Horticulture, Northwest A&F University, Yangling, Shaanxi, China; Key Laboratory of Biology and Genetic Improvement of Horticultural Crops (Northwest Region), Ministry of Agriculture, Yangling, Shaanxi, China; College of Life Sciences, Northwest A&F University, Yangling, Shaanxi, China; State Key Laboratory of Crop Stress Resistance and High-Efficiency Production, College of Horticulture, Northwest A&F University, Yangling, Shaanxi, China; Key Laboratory of Biology and Genetic Improvement of Horticultural Crops (Northwest Region), Ministry of Agriculture, Yangling, Shaanxi, China

## Abstract

Cold stress seriously affects plant growth and development. The ubiquitination system plays an important role by degrading and modifying substrates at the protein level. In this study, the U-box type ubiquitin ligase *VviPUB19* gene was induced by low temperature (4°C) in grapevine. In *Arabidopsis thaliana*, the *pub19* mutant, a homologous mutation of *VviPUB19*, exhibited enhanced cold tolerance, and the resistance phenotype of the mutant could be attenuated by *VviPUB19*. *VviPUB19-*overexpressing grape lines exhibited lower cold tolerance. Furthermore, it was revealed that VviPUB19 interacted with the cold-related transcription factors VviICE1, 2, and 3 and VviCBF1 and 2, and was involved in the degradation of them. This is the first time that an E3 ligase (VviPUB19) that interacts with CBFs and affects its protein stability has been identified. It was also shown that VviICE1, 2, and 3 positively regulated *VviPUB19* promoter activity. Therefore, our results suggest that VviPUB19 reduces grape cold tolerance via participating in the CBF-dependent pathway.

## Introduction

Cold stress (chilling stress and freezing stress) seriously affects plant growth and development. Plants have evolved a cold acclimation mechanism to cope with cold stress, in which plants gain greater tolerance to freezing through exposure to low non-freezing temperature [1, 2]. Studies have shown that a series of changes occurs in plants during this process, including accumulation of soluble sugars and protective proteins and induction of cold-responsive genes [1].

In *Arabidopsis thaliana*, CBFs/DREB1 include CBF1 (DREB1B), CBF2 (DREB1C), and CBF3 (DREB1A), which cause an increase in *COR* (*cold regulated*) gene expression, thus increasing the cold tolerance of plants [3, 4]. At the same time, CBF is also directly regulated by transcription factors, including the positive regulators ICE1 and 2 [5, 6] and CAMTA3 [7], and the negative regulators MYB15 [8] and EIN3 [9]. The stability of the bHLH-like transcription factor ICE1—as a key component of the cold response pathway—is crucial for plant cold response. ICE1 is modified by different proteins, such as ubiquitination of RING-type E3 ligase HOS1 [10]; SUMOylation of SIZ1 [11]; phosphorylation of OST1, MPK3/6, and BIN2 [12–14]; and DNA methylation [15]. Meanwhile, the transcriptional activity of ICE1 is regulated via interaction with the transcription factors MYB15, JASMONATE ZIM-DOMAIN (JAZ) and MYC67/70 [8, 16, 17]. In addition to Arabidopsis, ICE and CBF have been shown to be involved in cold stress in many plant species, such as grape [18–21].

Ubiquitination system finely regulates multiple biological processes in eukaryotes [22]. The components of the ubiquitination system include ubiquitin-activated enzyme (E1), ubiquitin-conjugating enzyme (E2), and ubiquitin ligase (E3) [23]. In Arabidopsis, there are >1400 E3 ligases [24]. In the process of ubiquitination, E3 ligase participates in binding substrates and is divided into four categories including U-box [24]. Plant U-box ubiquitin ligases (PUBs) contain a U-box domain of ~70 amino acids and were originally discovered in yeast [25]. Compared with yeast and humans [26, 27], the PUB family has shown obvious expansion in plants, including 64 members in Arabidopsis [28] and 56 members in grapes [29]. PUB proteins with a combination of U-box and ARM repeats comprise the largest category; furthermore, most of the PUB proteins with elucidated functions belong to this subcategory [30–32]. In Arabidopsis, PUB2 and PUB4 [33], PUB18/19 and PUB22/23 are involved in drought stress response; meanwhile PUB19 is also induced by cold [34, 35]; PUB25 and PUB26 positively regulate freezing resistance [36], and PUB12/13 participate in the immune response [37]. In grapevine, *Vitis amurensis* PUB (VaPUB) positively regulates the plant cold resistance [38, 39]; *Vitis pseudoreticulata* PUB24 (VpPUB24) enhances grape cold tolerance [40]; *Vitis labrusca* PUB38 (VlPUB38) negatively regulates fruit ripening [41].

Many studies have been reported on the cold tolerance of grapes. Firstly, the function of many genes involved in grape cold tolerance is verified, such as the kinase *VaCPK20* [42]; E3 ligase *VaPUB* [38] and *VpPUB24* [40]; transcription factors *VaICE1/2* [18], *VaCBF1* [43], *VaCBF4* [44], *VaERF057*, and *VaERF092* [45, 46]; and stress response gene *VaSAP15* [47] and *VaERD15* [48]. Then, the transcriptome, metabolome, and genomic technologies are used to screen out some genes that may be involved in the grape low-temperature pathway [49–51]. The advance of cold tolerance in grapes is reported in a recent review [52].

Grapes are a very nutritious economic commodity; however, cold stress limits their cultivation area and restricts the yield and quality. In Arabidopsis, the *PUB19* gene is induced by cold [34]. Our study showed that *VviPUB19* (Vitvi01g00782, XM_002264601), the homolog of Arabidopsis *PUB19* gene, was also induced by cold treatment. We then verified the cold-tolerant biological function of *VviPUB19* in Arabidopsis and grape and demonstrated that VviPUB19 interacted with the cold-related gene-encoding proteins VviICE1, 2, and 3 and VviCBF1 and 2, and participated in their degradation. It was also shown that VviICE1, 2, and 3 positively regulated *VviPUB19* promoter activity. So, it was suggested that VviPUB19 reduced the grape cold tolerance through the ICE–CBF–COR pathway, which further improved the knowledge regarding the molecular mechanism of grapevine response to cold stress.

## Results

### Cloning and expression analysis of ubiquitin ligase gene *VviPUB19* in grapevine

**Figure 1:**
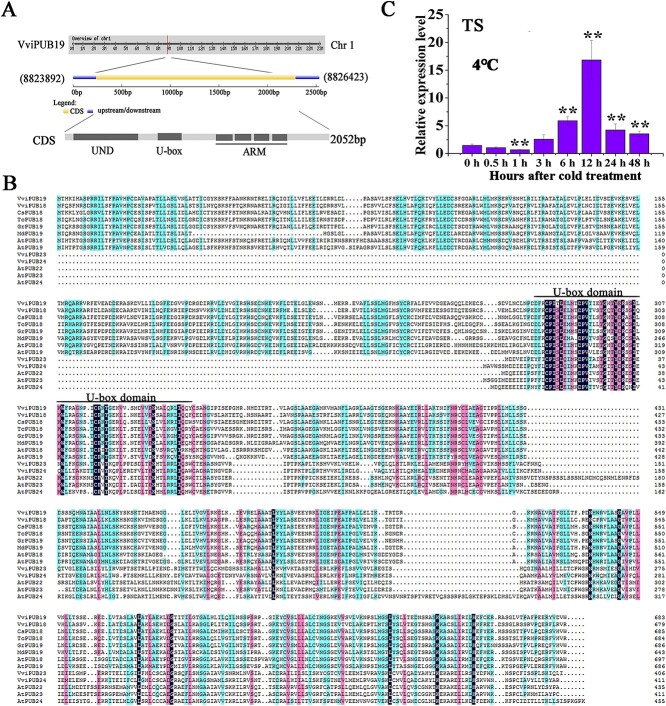
Characterization and expression analysis of ubiquitin ligase *VviPUB19.* A. Characterization analysis of the *VviPUB19* gene in grapevine. B. Multiple alignment analysis of the ubiquitin ligase VviPUB19. The black line represents the U-box domain. Gene numbers for multiple sequence alignment: VviPUB19 (XP_002264637.2), VviPUB18 (XP_002279989.1), CsPUB18 (XP_006483830.1), TcPUB18 (XP_007044546.2), GrPUB19 (XP_012479410.1), MdPUB19 (XP_008389629.3), AtPUB18 (At1g10560), AtPUB19 (At1g60190), VviPUB23 (NP_181137.2), VviPUB24 (XP_010651653), AtPUB22 (At3g52450), AtPUB23 (At2g35930), AtPUB24 (At3g11840). C. Expression analysis of *VviPUB19* in ‘Thompson Seedless’ at 4°C. All data are means ± SD of three biological replicates. Asterisk indicates significant difference (*t*-test, ^*^P < 0.05, ^**^P < 0.01).

In this study, the CDS (coding sequence) region of *VviPUB19* was amplified with the cDNA of ‘Thompson Seedless’ leaves as the template using *VviPUB19*-specific primers. The length of the *VviPUB19* gene was 2052 bp, encoding 682 amino acids, and located on chromosome 1 (Fig. 1a), and the GenBank accession number is OP485437. The GSDS analysis showed that the *VviPUB19* gene contained only one exon and no intron. Domain analysis indicated that VviPUB19 protein contained a UND domain, a U-box domain, and four repeated ARM domains (Fig. 1a). Multiple sequence alignment showed that VviPUB19, consistent with the PUB members in *Citrus sinensis*, *Gossypium raimondii*, *Theobroma cacao*, *Malus domestica*, and *A. thaliana*, contained the typical plant U-box domain of U-box ubiquitin ligase (Fig. 1a).

In our study, real-time quantitative PCR (RT-qPCR) indicated that *VviPUB19* was induced by 4°C in ‘Thompson Seedless’, and the expression of *VviPUB19* increased to a maximum level at 12 h (Fig. 1c). Meanwhile, we cloned the 2129-bp upstream promoter of the *VviPUB19* and found that it contained *cis-*acting elements related to cold response (Supplementary Fig. S1a). It was indicated that the *VviPUB19* promoter activity was induced by 4°C (Supplementary Figs. S1b and c).

### Complementation analysis of *VviPUB19* in *atpub19* mutants under cold stress

**Figure 2:**
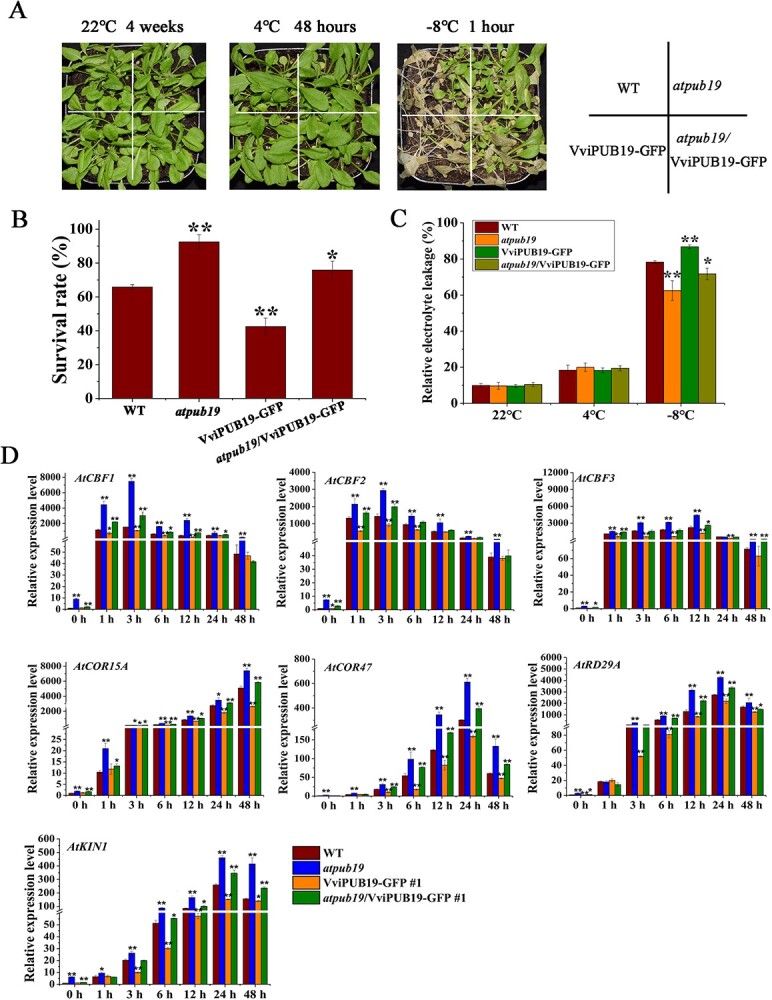
Functional analysis of *VviPUB19* and its orthologous gene *AtPUB19* in Arabidopsis under cold stress. A, B, and C. Analysis of phenotype (A), survival rate (B), and relative electrolyte leakage (C) of 4-week-old WT, *atpub19* mutant, *atpub19*/*VviPUB19-GFP* complementary line, and *VviPUB19-*overexpressing line under freezing treatment. For freezing treatment, different genotypes of Arabidopsis were first cold-acclimated at 4°C for 48 h, then cooled from 0°C to −8°C at a rate of 1°C h^−1^, then −8°C for 1 h. D. Expression analysis of cold-related genes in the WT, mutant, complementary line, and overexpressing line under the cold acclimated at 4°C. Data are means ± SD of three biological replicates. Asterisk indicates significant difference (*t*-test, ^*^P < 0.05 and ^**^P < 0.01).

Since the *PUB19* gene in grapevine was obviously induced with cold treatment, we validated the function in cold resistance. Firstly, the cold resistance of *VviPUB19* overexpressing Arabidopsis was analyzed (Supplemental Fig. S2a and b). The results of survival rates, relative electrolyte leakage, and cold-related gene expression analysis under cold stress showed that *VviPUB19-*overexpressing Arabidopsis lines exhibit lower cold tolerance (Supplemental Fig. S3). Then, the *atpub19* homozygous mutant and *atpub19/VviPUB19-GFP* complementary transgenic Arabidopsis (Supplementary Figs. S2c, d, and e) were also selected for cold resistance functional analysis. After cold stress treatment, the results of survival rates, relative electrolyte leakage, and cold-related gene expression (cold acclimated at 4°C) showed that the *atpub19* mutant and *VviPUB19-*overexpressing line had the highest and lowest cold tolerance, respectively, and the cold tolerance of complementary line was between them (Fig. 2). Thus, our results showed that the *VviPUB19* negatively regulated plant cold tolerance*.*

### *VviPUB19* reduces grapevine cold tolerance

**Figure 3:**
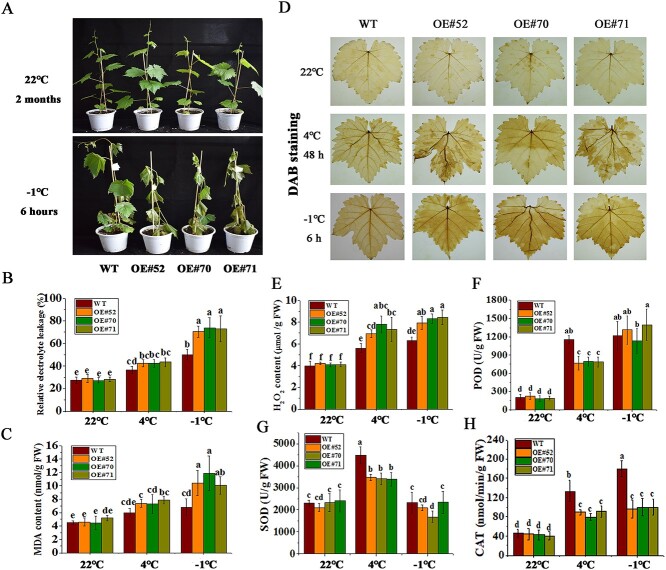
Evaluation of cold tolerance of *VviPUB19-*overexpressing grapes. The analysis of phenotypic (A), relative electrolyte leakage (B), MDA content (C), DAB strain (D), H_2_O_2_ content (E), POD activity (F), SOD activity (G), and CAT activity (H) after freezing treatment. The WT and transgenic grapes were grown at 22°C for 2 months and cold-acclimated at 4°C for 48 h, then treatment at −1°C for 6 h. All data are means ± SD of three biological replicates. Different letters indicate significant difference (Waller–Duncan test; *P* < 0.05).

*VviPUB19* gene was also overexpressed in ‘Thompson Seedless’, and OE#52, OE#70, and OE#71 lines were selected for cold tolerance validation (Supplemental Fig. S4). After the freezing treatment, it was shown that three transgenic lines had obvious freezing damage phenotype of wilting leaves, while the leaves of the wild type (WT) were only slightly wilted (Fig. 3a), indicating that the *VviPUB19-*overexpressing grape plants had reduced freezing resistance.

After cold acclimation at 4°C and treatment at −1°C, the *VviPUB19-*overexpressing plants had higher relative electrolyte leakage and malondialdehyde (MDA) content (Fig. 3b and c). Under 4°C and −1°C treatment, diaminobenzidine (DAB) staining analysis showed that the leaves of the overexpressing lines were more deeply stained (Fig. 3d), and the H_2_O_2_ content analysis indicated that the H_2_O_2_ levels of overexpressing plants were higher (Fig. 3e). Furthermore, the transgenic plants had lower peroxidase (POD), superoxide dismutase (SOD), and catalase (CAT) activities after treatment at 4°C (Fig. 3f–h). This suggested that the *VviPUB19-*overexpressing grapes had lower cold tolerance.

### Overexpression of *VviPUB19* inhibits the expression of cold-responsive genes

**Figure 4:**
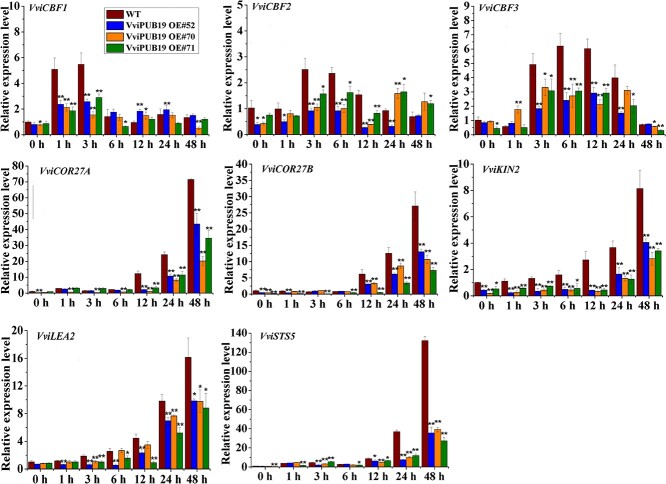
Expression of cold-responsive genes in *VviPUB19-*overexpressing plants under treatment at 4°C. WT and *VviPUB19-*overexpressing plants were harvested after 0–48 h at 4°C treatment. Data are means ± SDs of three biological replicates. Asterisks represent significant differences (*t*-test, ^*^P < 0.05 and ^**^P < 0.01).

Then the induction of cold-responsive genes in the WT and the *VviPUB19*-overexpressing plants was detected using RT-qPCR (Fig. 4). Compared to the WT, the induction degree of *VviCBF1* and *VviCBF2*/*3* gene in the three overexpressing plants was significantly lower after 1–3 and 3–12 h at 4°C, respectively; the expression of the *VviCOR27A*, *VviCOR27B*, *VviLEA2*, and *VviSTS5* genes was downregulated in the overexpressing lines after 12–48 h of treatment at 4°C; the expression of *VviKIN2* was always significantly lower in the overexpressing plants than in the normal conditions and cold treatment.

### VviPUB19 interacts with VviICE1, 2, and 3 and mediates their degradation

**Figure 5:**
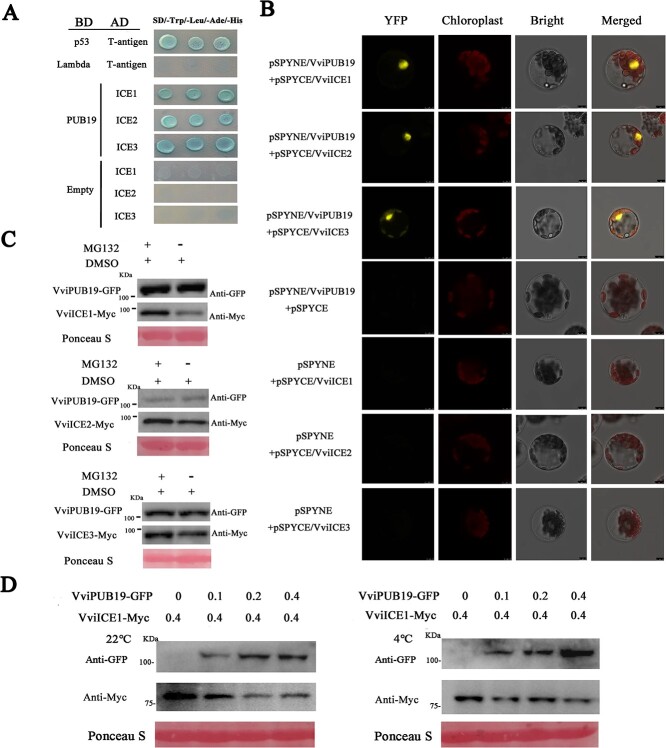
Interaction and protein degradation analysis of VviPUB19 with VviICE1, 2, and 3. A. VviPUB19 interacts with VviICE1, 2, and 3 in the Y2H. A different prey vector and bait vector were used to co-transform Y2H yeast cells and verify the interaction between proteins on the medium containing 200 ng/ml AbA. B. BiFC assay verify the interaction between VviPUB19 and VviICE1, 2, and 3. The plasmids were co-transformed into protoplasts, and YFP fluorescence was observed after culture in darkness for 20 h. C. MG132 inhibits the degradation of VviICE1, 2, and 3 by VviPUB19. After co-transformation for 24 h, DMSO or 50 μM MG132 + DMSO was injected, and then cultured for 36 h before sampling. D. VviPUB19 participates in the degradation of VviICE1 at 22°C and 4°C. *Agrobacterium* carrying different concentrations of the VviPUB19-GFP + VviICE1-Myc combination were injected into different parts of the same leaf. At 22°C, samples were taken directly after co-transformation for 60 h; for the 4°C treatment, samples were taken at 4°C for 6 h after co-transformation for 60 h. The numbers 0, 0.1, 0.2, and 0.4 represent the concentration of *Agrobacterium* at OD_600_.

In this study, we found that *VviPUB19* was induced by cold stress, and overexpression of *VviPUB19* reduced the expression of *CBF genes*, which are downstream of the transcription factor ICEs. Therefore, we speculated that VviPUB19 may participate in the cold stress response of grapes by interacting with VviICE1 (XM_010658531), VviICE2 (XM_002275675), and VviICE3 (XM_002284492). The yeast two-hybrid (Y2H) results showed that yeast cells co-transformed with VviICE1, 2, and 3 and VviPUB19 grew normally, indicating that VviPUB19 interacts with VviICE1, VviICE2, and VviICE3 (Fig. 5a). Then we further verified that the UND and ARM domains of VviPUB19 interacted with VviICE1, VviICE2, and VviICE3 (Supplemental Fig. S5a). In addition, we found that AtPUB19 interacted with AtICE1 and AtICE2 in Arabidopsis (Supplemental Fig. S6a).

Next, the bimolecular fluorescence complementation (BiFC) assay to further determine VviPUB19 interacts with VviICEs. pSPYNE/VviPUB19 and pSPYCE/VviICE1, 2, and 3 recombinant plasmids were co-transformed into Arabidopsis protoplasts. The results indicated that YFP fluorescent signal was only displayed in the combinations of pSPYNE/VviPUB19 + pSPYCE/VviICE1, 2, and 3, but not in controls (Fig. 5b). At the same time, we also verified that the UND and ARM domains of VviPUB19 interacted with VviICE1, VviICE2, and VviICE3 via BiFC assay (Supplemental Fig. S5b).

To analyze whether the ubiquitin ligase VviPUB19 degrades VviICE1, 2 and 3, we performed a degradation assay *in vivo*. Firstly, we found that the proteasome inhibitor MG132 is involved in the influence of VviPUB19 on the expression of VviICE1, 2, and 3 proteins (Fig. 5c). Then, VviICE1 was selected for gradient degradation testing. Western blot analysis showed that VviPUB19-GFP protein inhibited the protein content of VviICE1-Myc at 22°C and 4°C (Fig. 5d). Therefore, our findings suggested that VviPUB19 degraded VviICE1, 2, and 3 proteins and was inhibited by MG132.

### VviPUB19 interacts with VviCBF1 and 2 and mediates their degradation

**Figure 6:**
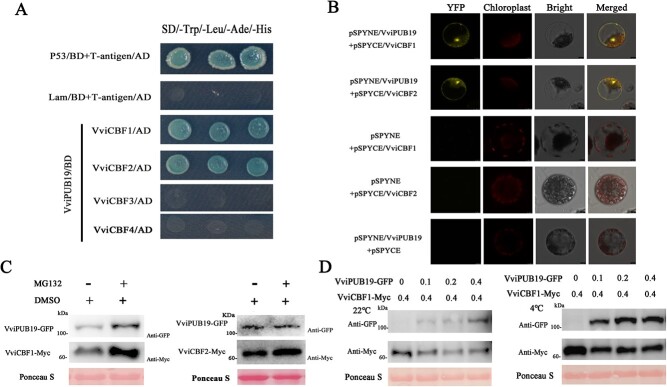
Interaction and protein degradation analysis of VviPUB19 with VviCBF1 and VviCBF2. A. VviPUB19 interacts with VviCBF1 and VviCBF2 in a Y2H assay. A different prey vector and bait vector were used to co-transform Y2H yeast cells and verify the interaction between proteins on the medium containing 200 ng/ml AbA. B. VviPUB19 interacts with VviCBF1 and VviCBF2 in a BiFC assay. The plasmids were transformed into protoplasts, and the YFP fluorescence was observed after culture in darkness for 20 h. C. MG132 inhibits the degradation of VviCBF1 and VviCBF2 by VviPUB19. After co-transformation for 24 h, DMSO or 50 μM MG132 + DMSO was injected, and then cultured for 36 h for sampling. D. Degradation analysis of VviCBF1 by VviPUB19 in tobacco at 22°C and 4°C. *Agrobacterium* carrying different concentrations of the VviPUB19-GFP + VviCBF1-Myc combination were injected into different parts of the same leaf. For 22°C, samples were taken directly after co-culturing for 60 h. For the 4°C treatment, samples were taken at 4°C for 6 h after co-transformation for 60 h. The numbers 0, 0.1, 0.2, and 0.4 represent the concentration of *Agrobacterium* at OD_600_.

In plant cold tolerance, the transcription factor CBFs may play a more important role than ICEs, because they can directly regulate the expression of *COR* genes; therefore, we also analyzed the interaction between VviPUB19 and VviCBF1 (KF582113), VviCBF2 (AY390376), VviCBF3 (KF758762), and VviCBF4 (XM_019226062). The results of Y2H indicated that VviPUB19 interacted with VviCBF1 and VviCBF2 (Fig. 6a) and only the ARM domain of VviPUB19 interacted with VviCBF1 or VviCBF2 (Supplemental Fig. S7). Meanwhile, we also verified that AtPUB19 can interact with AtCBF1 and AtCBF2 (Supplemental Fig. S6b). We then determined the interaction between VviPUB19 and VviCBF1 or VviCBF2 via BiFC assay. The results indicated that YFP fluorescent signal was only displayed in the protoplasts of co-transformed pSPYNE/VviPUB19 and pSPYCE/VviCBF1 or pSPYCE/VviCBF2 (Fig. 6b).

To further analyze their degradation relationship, a degradation assay *in vivo* was performed. Firstly, we found that MG132 is involved in inhibiting the degradation of VviCBF1 and VviCBF2 protein content by VviPUB19 (Fig. 6c). Then, VviCBF1 was selected for gradient degradation testing. Western blot analysis showed that VviPUB19-GFP protein inhibited the protein content of VviCBF1-Myc at 22°C and 4°C (Fig. 6d). Therefore, our findings suggested that VviPUB19 is involved in the degradation of VviCBF1 and VviCBF2 protein and is inhibited by MG132.

### Transcription factor VviICEs positively regulate the activity of the *VviPUB19* promoter

**Figure 7:**
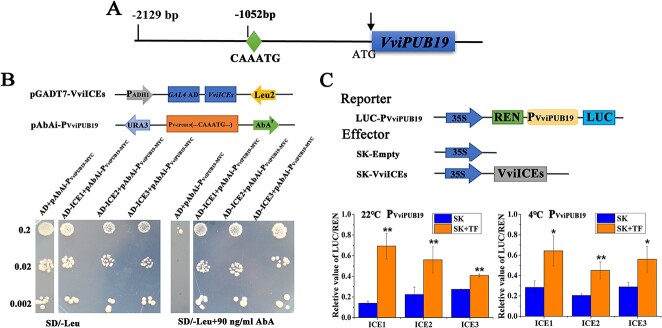
The transcription factors VviICE1, 2, and 3 interact with *VviPUB19* promoter activity. A. Location diagram of the MYC element (CAAATG) on the *VviPUB19* promoter. B. The interaction between VviICE1, VviICE2, or VviICE3 and the *VviPUB19* promoter was verified via Y1H assay. The interaction was verified on SD/−Leu medium containing 90 ng/ml AbA. C. Analysis of regulation of VviICE1, VviICE2, and VviICE3 on the activity of the *VviPUB19* promoter at 22°C and 4°C. For 22°C, samples were taken directly after co-culturing for 60 h. For the 4°C treatment, samples were taken at 4°C for 6 h after co-culturing for 60 h. The co-transformation of 62-SK and the *VviPUB19* promoter was used as control. All data are means ± SD of three biological replicates. Asterisk indicates significant difference (*t*-test, ^*^P < 0.05 or ^**^P < 0.01)

Due to the *VviPUB19* promoter (P_VviPUB19_) containing the ICE-binding element MYC and CBF-binding element DRE (Fig. 7a and Supplemental Fig. S1a), the interaction between ICE and CBF transcription factors and the *VviPUB19* promoter in grapevine was verified via yeast one-hybrid (Y1H) assay. It was found that 90 ng/ml AbA inhibited the growth of AD+pABAi-P_VviPUB19-MYC_ co-transformed colonies on SD/−Leu medium, but did not inhibit the growth of AD-VviICE1 + pABAi-P_VviPUB19-MYC_, AD-VviICE2 + pABAi-P_VviPUB19-MYC_, and AD-VviICE3 + pABAi-P_VviPUB19-MYC_ co-transformed colonies (Fig. 7b). Therefore, our results showed that VviICE1, VviICE2, and VviICE3 interacted with the *VviPUB19* promoter. Unfortunately, the VviCBF1, VviCBF2, VviCBF3, and VviCBF4 transcription factors failed to bind to the DRE element of the *VviPUB19* promoter (data not shown).

A dual-luciferase assay was performed to analyze the regulation of *VviPUB19* promoter activity by the transcription factors VviICE1, VviICE2, and VviICE3. The results showed that the LUC/REN ratio of co-transformed SK-VviICE1 + LUC-P_VviPUB19-MYC_, SK-VviICE2 + LUC-P_VviPUB19-MYC_, and SK-VviICE13 + LUC-P_VviPUB19-MYC_ was significantly higher than that of co-transformed SK + LUC-P_VviPUB19-MYC_ at either 22°C or 4°C (Fig. 7c). Therefore, VviICE1, VviICE2, and VviICE3 significantly enhanced *VviPUB19* promoter activity at both 22°C and 4°C, and then positively regulated *VviPUB19* gene expression.

## Discussion

To date, many studies related to the cold-tolerance mechanisms of grape have been carried out [46, 51, 53]. There are few studies on the PUB in grapes under cold stress; only the functions of VaPUB and VpPUB24 have been characterized [38, 40] and our published article showed that VviPUB19 is involved in the degradation of VviERF105, which promotes tolerance to abiotic stress, including cold [54]. Both the gene expression and promoter activity of *VviPUB19* were induced by cold treatment (Fig. 1 and Supplemental Fig. S1), so we investigated the function and molecular mechanism of *VviPUB19* in cold stress.

Firstly, the function of the *VviPUB19* gene in cold tolerance was validated in the model plant Arabidopsis. It was found that the freezing resistance of the *atpub19* mutant was enhanced, and the freeze-resistant phenotype could be complemented by *VviPUB19* (Fig. 2). Then, we also found that *VviPUB19-*overexpressing grapes had reduced freezing resistance (−1°C, 6 h) (Fig. 3a), and the expression of *CBFs* and *COR27A*, *COR27B*, *LEA2,* and *STS5* [53] in transgenic lines was significantly lower after cold treatment (Fig. 4). In conclusion, our study indicated that *VviPUB19* and its orthologous gene *AtPUB19* negatively regulated cold tolerance by influencing related physiological indices and gene expression.

Our study found that VviPUB19 interacted with VviICE1, 2, and 3 and mediated their degradation, which in turn negatively regulated grape cold tolerance. Due to the fact that VviICE1, 2, and 3 in grapes belong to paralogous genes, they share the same structural domain and similar sequences. So, when VviPUB19 interacts with VviICE1, it also interacts with the other two proteins. Our previous study also showed that PUB24 and HOS1 interacted with ICE1 in grapevine [40]. The VviICE1 protein contains three domains: bHLH, ZIP, and ACT. Since all the three ubiquitin ligases PUB19, PUB24, and HOS1 interact with the ICE1 protein in grapevine, we further analyzed the interaction between the domains of VviICE1 and them. The Y2H results showed that only the ACT domain was involved in the interaction with VviPUB19, VviPUB24, and VviHOS1 (Supplemental Fig. S8). Meanwhile, previous studies have also shown that the ACT domain of ICE1 interacts with PUB24 or JAZ [16, 40]. The finding that the ACT region of VviICE1 mediates its interaction with VviPUB19, VviPUB24, and VviHOS1 led us to ask whether the three ubiquitin ligases display a competitive, cooperative, or redundant effect on the physical association with VviICE1; it is very meaningful to compare the affinity between VviICE1 and the three ubiquitin ligases.

It is well known that ICE transcription factors are induced to express at low temperature. Our study not only showed that VviPUB19 mediated the degradation of the VviICE1, 2, and 3 proteins, but also demonstrated that the VviICE1, 2, and 3 transcription factors directly positively regulated *VviPUB19* promoter activity to increase its gene expression (Fig. 7). This cyclic relationship led us to speculate that there might be a balance in regulation between VviPUB19 and VviICE1 at low temperature: the expression of the *VviICE1* transcription factor was greatly induced at low temperature, and the promoter activity and gene expression of the *VviPUB19* in plants were activated by VviICE1, which resulted in the increased expression of the VviPUB19 protein. At this time, the expression of the VviPUB19 protein accelerated the degradation of the VviICE1, so that it would not always be in the activated state and stayed at a low level. Therefore, we believe that VviPUB19 and VviICE1 affect the process of plant response to low temperature stress via mutual regulation at the gene and protein levels.

At present, there have been many studies on the regulation of the transcription level of CBF transcription factors, while their regulation at the translational and post-translational levels remains unclear. Under low temperature, 14–3-3λ proteins are phosphorylated by kinase CRPK1 and interacting with CBF, then degrade it through the 26S proteasome pathway [55]. However, the E3 ligases that directly interact with the CBF protein and ubiquitination degradation have not been reported. In this study, we found that VviPUB19 interacted with VviCBF1 and VviCBF2 and participated in their degradation *in vivo* (Fig. 6). We also conducted *in vitro* ubiquitination assay to demonstrate the ubiquitination relationship between VviPUB19 and VviCBF1, but found that VviPUB19 does not seem to be involved in the *in vitro* ubiquitination of VviCBF1. We speculate that there may be other proteins activated or involved in the ubiquitin process *in vivo*, while *in vitro* assay cannot simulate the real situation *in vivo*. Previous study have also shown that OsSOR1 interacts with OsIAA9 and contributes to its degradation, but OsSOR1 cannot ubiquitinate OsIAA9, meaning that this degradation relationship does not require the E3 ubiquitin ligase activity of OsSOR1 [56]. Anyway, this is the first time that an E3 ligase that interacts with CBF and affects its protein stability has been identified. Meanwhile, Y2H results showed that VviPUB19 did not interact with VviCBF3 and VviCBF4 (Fig. 6a). However, it is unclear why PUB19 interacted only with CBF1 and 2 but not with CBF3 and 4, which may require further studies to verify.

There are 64 PUB encoded in Arabidopsis; among these, 17 members of the PUB-ARM class contain an N-terminal UND motif [32]. The ARM repeat motifs and UND domain have been shown to be involved in physical interactions with proteins [35, 57]. The UND domain determines that AtPUB18/AtPUB19 and AtPUB22/AtPUB23 reduce drought resistance through ABA dependent and independent pathways, respectively [34, 35]. This indicates that the UND domain plays an important role in the biological functions of PUB proteins. In our study, the ARM domain interacted with VviCBF1 and 2 (Supplemental Fig. S7), and both UND and ARM domains interacted with VviICE1, 2, and 3 (Supplemental Fig. S5). Therefore, we believe that the N-terminal UND domain may play different roles when VviPUB19 interacts with different substrate proteins.

The expression levels of ICE and CBF transcription factors are very low in plants in a normal environment, and they are greatly induced to express when plants are subjected to stresses, thus activating downstream resistance genes and enhancing plant tolerance. The expression will not always be activated and will return to the preinduction level in a certain period of time, which may be due to the plant growth, and development will be affected when the resistance genes are expressed at a high level for a long time. It has been found that overexpression of ICE and CBF transcription factors seriously affect the growth and development of plants [5, 58, 59]. So, it is an important scientific issue to explore the balance between plant growth and development and stress resistance. Our study found that VviPUB19-overexpressing plants grew normally and had reduced cold tolerance; then, it was found that VviPUB19 was involved in the degradation of VviICE1 and VviCBF1 at 22°C and 4°C. Therefore, we speculated that VviPUB19 may maintain plant growth in the natural environment by inhibiting the expression of VviICE1 and VviCBF1, and reduce plant cold tolerance by degrading VviICE1 and VviCBF1 under cold stress.

## Conclusion

**Figure 8:**
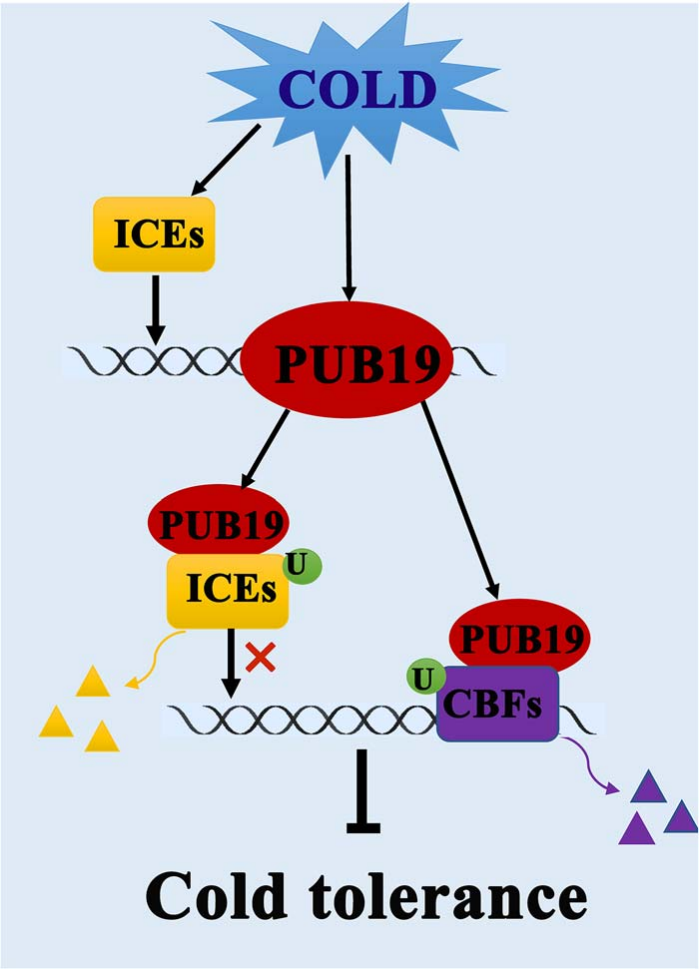
A model of VviPUB19 negatively regulates grape cold tolerance. Low temperature induces *ICEs* gene expression, which activates the activity of *PUB19* promoter to increase its gene expression. Then, the PUB19 protein interacts with ICEs and participates in their ubiquitination degradation, thereby inhibiting the gene expression of downstream CBFs of ICEs. Meanwhile, PUB19 protein interacts with CBFs and participates in their ubiquitination degradation, thereby directly inhibiting CBFs protein expression. Therefore, PUB19 negatively regulates grape cold tolerance via the ICE-CBF-COR pathway. The ‘U’ represents ubiquitin, and the different colored triangles represent degraded protein.

*VviPUB19* was induced by 4°C and reduced Arabidopsis and grape cold tolerance, and the cold resistance phenotype of the *pub19* mutant could be complemented by *VviPUB19*. Furthermore, it was revealed that VviPUB19 interacted with VviICE1, 2, and 3 and VviCBF1 and 2, and was involved in the degradation of them. It was also shown that VviICE1, 2, and 3 positively regulated *VviPUB19* promoter activity. Here is a model to represent the negative regulation of grape cold tolerance by VviPUB19 (Fig. 8).

## Materials and methods

### Plant materials

‘Thompson Seedless’ cuttings grown in pots (diameter 28 cm, height 14 cm) in the greenhouse (temperature 22°C–30°C; humidity 70%–93%) of Northwest A&F University in Yangling, Shaanxi, China. The *Nicotiana tabacum* and *A. thaliana* were grown in a phytotron (temperature 18°C–22°C; humidity 70%). The T-DNA mutant *atpub19* (SALK_152677) was obtained from the AraShare Technology Service Center (https://www.arashare.cn/index/Product/index.html), and screen for *pub19* homozygous mutants by detection of DNA and RNA (Supplemental Fig. S2, c and d).

### Bioinformatics analysis

The exons and introns of gene were analyzed using the gene structure display server website (GSDS, http://gsds.gao-lab.org/). The domain was identified using SMART (http://smart.embl-heidelberg.de/) and the Conserved Domains Database (NCBI; http://www.ncbi.nlm.nih.gov/Structure/cdd/cddsrv.cgi). Multiple sequence alignment analysis was performed using DNAMAN (Lynnon Biosoft, USA) software.

### Low-temperature treatment

Cold treatment of 4°C: When the ‘Thompson Seedless’ grape cuttings grew in a greenhouse for 4 months and have eight leaves, we selected six healthy seedlings for cold treatment. Before cold treatment, potted seedlings were transferred from the greenhouse to a phytotron (22°C) for 3 days and then treated with incubation at 4°C (low-temperature incubator). Leaves were collected at 0–48 h after the 4°C treatment. Meanwhile, the leaves at 22°C were used as the control at the corresponding time. Three leaves from the three different plants were collected as biological replicates and were then stored at −80°C.

Freezing treatment of Arabidopsis: Select 4-week-old plants to be cold-acclimated at 4°C for 48 h, then cooling at a rate of 1°C h^−1^ from 0°C to −6°C, −7°C, or − 8°C, then continue for 1 h (low-temperature incubator). Seedlings were overnight at 4°C, and then grew at 22°C for 5 days to calculate the survival rate. The relative conductivity was measured after treatment at −8°C and the cold response expression analysis was conducted at 4°C for 0–48 h.

Freezing treatment of grape: Select 2-month-old WT and *VviPUB19* transgenic grapes to be cold-acclimated at 4°C for 48 h, then treatment at −1°C for 6 h (low-temperature incubator). After the freezing treatment, phenotype and physiological and biochemical index analyses were conducted. The expression analysis of cold-responsive genes was conducted at 4°C for 0–48 h.

### Measurements of physiological indices and 3,3′-diaminobenzidine staining

The relative electrolyte leakage analysis was performed using a previous method [54]. The contents of MDA and hydrogen peroxide (H_2_O_2_) as well as activities of POD and SOD were measured using the visible spectrophotometry kit (Comin Biotechnology, China). CAT activity was determined using the UV spectrophotometry kit (Comin Biotechnology, China). The samples used for the determination of physiological indices were plant fresh leaves with a weight of 100 mg, and each treatment was performed with three biological replicates from three independent leaves. Three grape leaves treated at 22°C and low temperature (4°C and −1°C) were selected for 3,3′-DAB staining, referring to the previous method [60].

### RT-qPCR assay

RNA extraction and cDNA synthesis were performed using the EZNA Plant RNA Kit (Omega, USA) and the cDNA Synthesis SuperMix (Transgen, China), respectively. RT-qPCR was performed using SYBR (Transgen, China) and QuanStudio 6 Flex system (Applied Biosystems, USA). *VviActin* or *AtActin* was used as an internal reference. The relative expression was determined using the 2^-ΔΔCT^ method [61]. The gene IDs and primers for RT-qPCR analysis are shown in Supplemental Table S1.

### GUS activity analysis

DNA was extracted using the modified CTAB method [62] and used as the template to clone the *VviPUB19* promoter. Then, The *VviPUB19* promoter was connected to the pCAMBIA0390: GUS vector to construct the P_VviPUB19_-GUS fusion expression vector using seamless cloning technology for promoter activity analysis (primers in Supplemental Table S2). *Agrobacterium* containing different GUS vectors was transitively transformed into tobacco, cultured for 48 h, then sampled after treatment at 4°C for 6 h. The GUS staining and GUS quantitative analysis were carried out using our previous methods [63].

### Plant transformation

The *VviPUB19* gene was ligated into pCAMBIA2300-GFP to construct the overexpression vector 35S-VviPUB19-GFP (primers in Supplemental Table S2). The genetic transformation of Arabidopsis was performed using the inflorescence dip method [64]. The genetic transformation of ‘Thompson Seedless’ grape was carried out using *Agrobacterium-*mediated organogenesis [65], and screening on standard MS medium containing 50 mg/l kanamycin+300 mg/l Cephalosporin+200 mg/l Carbenicillin, then the resistant callus tissue was induced into seedlings through organ regeneration pathways.

### Y2H assay

The *VviPUB19* and fragments of different deletion domains were inserted into the pGBKT7 (BD) vector to construct the bait vectors BD-VviPUB19, BD-VviPUB19^∆U-box + ARM^, BD-VviPUB19^∆UND + ARM^, and BD-VviPUB19^∆UND + U-box^. The *VviICE1*/*VviICE2*/*VviICE3*/*VviCBF1*/*VviCBF2*/*VviCBF3*/*VviCBF4* gene was cloned and inserted into pGADT7 (AD) to construct the prey vector (primers in Supplemental Table S2). Y2HGold yeast strains were co-transformed with the prey and bait plasmids and then on SD/−Trp/−Leu/−Ade/-His/x-α-Gal+200 ng/ml aureobasidin A (AbA) to verify protein interactions. The bait vector autoactivation assay showed that the concentration of AbA that inhibited the self-activation of BD-VviPUB19 was 200 ng/ml.

### BiFC assay

For the BiFC assay, the *VviPUB19* and fragments of different deletion domains were ligated to pSPYNE vector to construct the fusion vectors pSPYNE/VviPUB19, pSPYNE/VviPUB19^∆U-box + ARM^, and pSPYNE/VviPUB19^∆UND + U-box^. The *VviICE* and *VviCBF* genes were inserted into the pSPYCE vector to generate pSPYCE/VviICE1, pSPYCE/VviICE2, pSPYCE/VviICE3, pSPYCE/VviCBF1, and pSPYCE/VviCBF2 (primers in Supplemental Table S2). Different combinations of fusion vectors were co-transformed into Arabidopsis protoplasts to analyze the interaction between proteins. The laser confocal microscopy was used to observe YFP fluorescence (TCS SP8 SR, Leika, Germany).

### Protein degradation assay

Protein degradation assay used a previously reported method [54]. The CDS of VviPUB19-interacting proteins was inserted into pCAMBIA1307-Myc to generate various 35S-Myc fusion vectors (primers in Supplemental Table S2). Then, various 35S-Myc fusion vectors and 35S-VviPUB19-GFP combinations were transiently co-transformed into tobacco leaves with different ratios of the *Agrobacterium* cultures (0, 0.1, 0.2, and 0.4). For the 22°C treatment, samples were directly collected after being co-cultured for 60 h; for the 4°C treatment, infiltrated tobacco was co-cultured at 22°C for 60 h and samples were harvested after 6 h at 4°C. For proteasome inhibitor MG132 treatment, DMSO or 50 μM MG132 + DMSO was injected to leaves. The proteins were extracted with Western & IP Lysis Buffer (Coolaber, China) for western blot analysis [66].

### Ubiquitination assay

To conduct *in vitro* ubiquitination assay, the *VviPUB19* and *VviCBF1* were inserted into the vector pMAL-c5X and pCold ProS2 DNA, respectively, to construct fusion expression vectors VviPUB19-MBP and VviCBF1-His (primers in Supplemental Table S2). Then the protein was induced at 16°C for 20 h, and purified recombinant proteins of VviPUB19-MBP and VviCBF1-His were obtained using pMAL™ Protein Fusion & Purification System (NEB #E8200S) and NI-NTA Beads (Smart-Lifesciences Biotech), respectively.

*In vitro* ubiquitination assay was performed according to the instructions of the ubiquitin kit (UB biotech). The reaction system was 20 μl: 2 μl 10 × reaction buffer, 2 μl 10 × E1 enzyme, 1 μl 20 × E2 enzyme, 4 μl 5 × ubiquitin, 2 μl 10 × Mg^2+^-ATP, 2 μl VviPUB19-MBP, 2 μl VviCBF1-His, and 5 μl H_2_O. The mixture was then incubated at 37°C for 4 h, followed by adding 5 μl of 5 × SDS loading buffer and boiled for 5 min for western blot analysis. Anti-MBP (TransGen Biotech), anti-His (TransGen Biotech), and anti-ubiquitin (cell signaling technology) antibodies were used to detect proteins, respectively.

### Y1H assay

*Cis*-acting element analysis indicated that the *VviPUB19* promoter contains MYC and DRE elements. Then, the MYC or DRE fragment of the *VviPUB19* promoter was inserted into the pAbAi vector to construct pAbAi bait vector (primers in Supplemental Table S2). Subsequently, Y1H assay was used to determine whether grapevine ICE and CBF transcription factors bound the *VviPUB19* promoter. The Y1HGold yeast strain containing pABAi-P_VviPUB19-MYC_ or pABAi-P_VviPUB19-DRE_ was used for the transformation of AD-VviICE1, 2, and 3 or AD-VviCBF1, 2, 3, and 4, and then validate the interaction between transcription factor and promoter on SD/− Leu + AbA medium.

### Dual-luciferase assay

The *VviICE1*, *VviICE2*, and *VviICE3* genes were inserted into the pGreenII 62-SK vector and the MYC fragment of the *VviPUB19* promoter was cloned into the pGreenII 0800-LUC vector (primers in Supplemental Table S2). The SK-VviICE1, SK-VviICE2, or SK-VviICE3 and LUC-P_VviPUB19-MYC_ were co-transformed into tobacco. For the 22°C treatment, samples were taken directly after co-culturing for 60 h. For the 4°C treatment, samples were taken at 4°C for 6 h after co-culturing for 60 h. The activity of luciferase was assayed using a dual-luciferase reporter gene detection kit (Yisheng, China). The LUC/REN value indicated the promoter activity. The empty vector 62-SK was co-transformed with LUC-P_VviPUB19-MYC_ as a control.

### Statistical analysis

The statistical analysis was performed by one-way ANOVA using the SPSS Statistics 21.0 software and Student’s *t*-test (http://www.physics.csbsju.edu/stats/t-test_bulk_form.html), significant differences are indicated by different letters (Waller–Duncan’s test; *P* < .05) and asterisks (*t*-test; * *P* < .05, ** *P* < .01).

## Supplementary Material

Web_Material_uhae297

## Data Availability

The data that support the results are provided in this paper and its supplementary files.
