# Supplementary material for: The ubiquitin ligase VviPUB19 negatively regulates grape cold tolerance by affecting the stability of ICEs and CBFs
Source: Hortic Res. 2024 Oct 23;12(2):uhae297. doi: 10.1093/hr/uhae297 (PMC11822393; doi:10.1093/hr/uhae297)
Supplement: Web_Material_uhae297 [file web_material_uhae297.zip › Supplementary figures.docx]

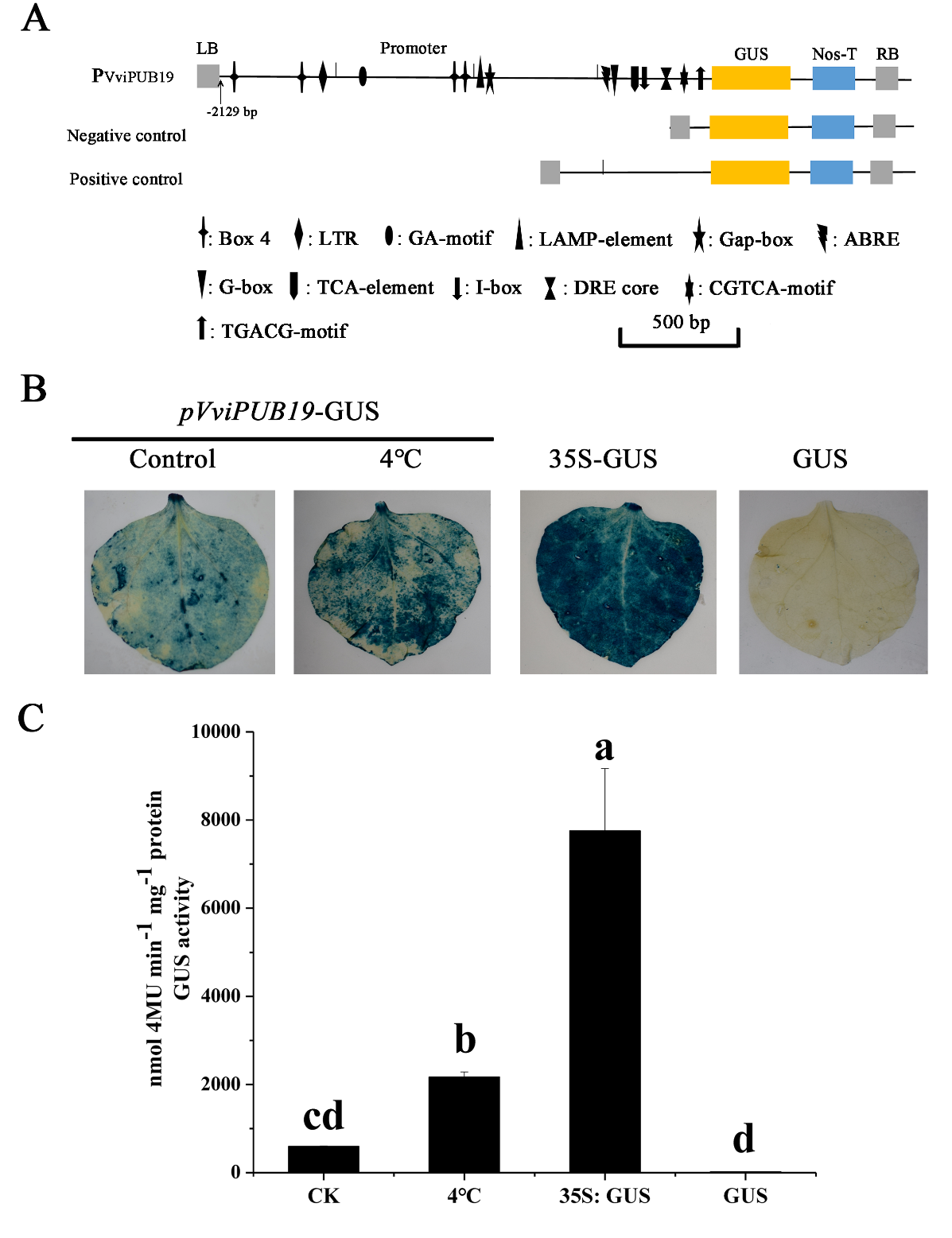


**Fig. S1.** **Analysis of *cis*-acting elements and GUS activity of *VviPUB19* promoter.** **A.** Schematic diagram of the *cis*-acting element of the *VviPUB19* promoter (-2129 bp). Light responsive elements: Box 4, GA-motif, LAMP-element, Gap-box, G-box and I-box; Low temperature responsive element: LTR; ABA responsive element: ABRE; SA responsive element: TCA-element; CBF binding element: DRE core; MeJA responsive elements: CGTCA-motif and TGACG-motif. **B and C.** Analysis of GUS activity of *VviPUB19* promoter under 4℃ treatment. *Agrobacterium*-mediated transiently transformed tobacco leaves were cultured for 48 h, then treated under 4℃ for 6 h and sampled for GUS staining (B) and GUS quantitative analysis (C). The experiments were performed in three biological replicates. Significant differences are indicated with different letters (Waller-Duncan test; *P* < 0.05).


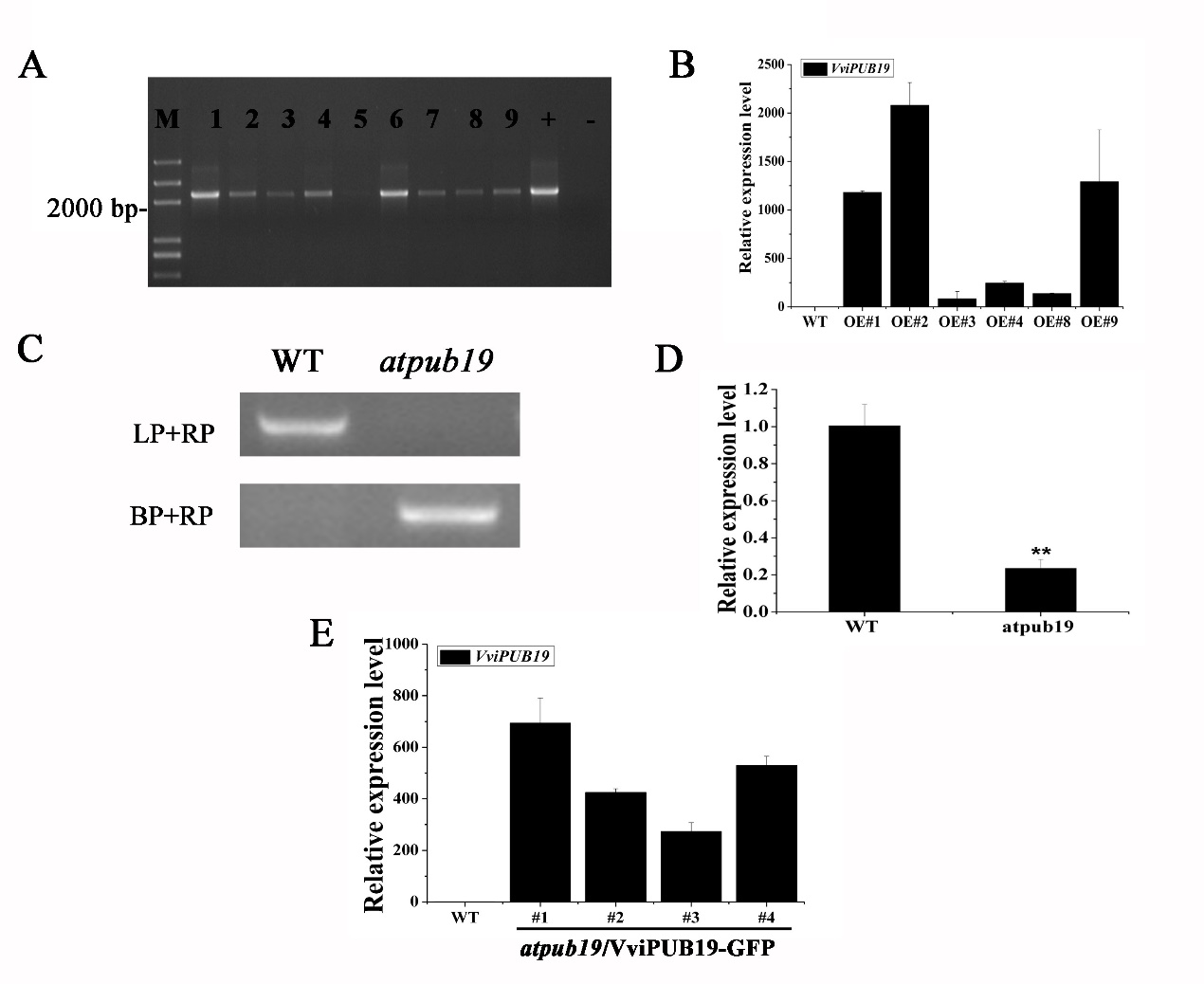


**Fig. S2.** **Detection of overexpression of *VviPUB19* or mutation of *AtPUB19* in *Arabidopsis thaliana*. A.** DNA detection of *VviPUB19* transgenic Arabidopsis, Lane M: Marker, Lane 1-9: different *VviPUB19* overexpressing lines, Lane +: positive control, Lane -: WT; **B.** Detection of RNA levels in three *VviPUB19* overexpressing lines. **C and D.** Detection of DNA (C) and RNA (D) in Arabidopsis *pub19* mutants. **E.** Detection of RNA levels of *VviPUB19* complementary lines. Values shown are mean ± SD from three biological replicates. Significant differences are indicated with an asterisk (*t*-test, **P* < 0.05 or ***P* < 0.01).


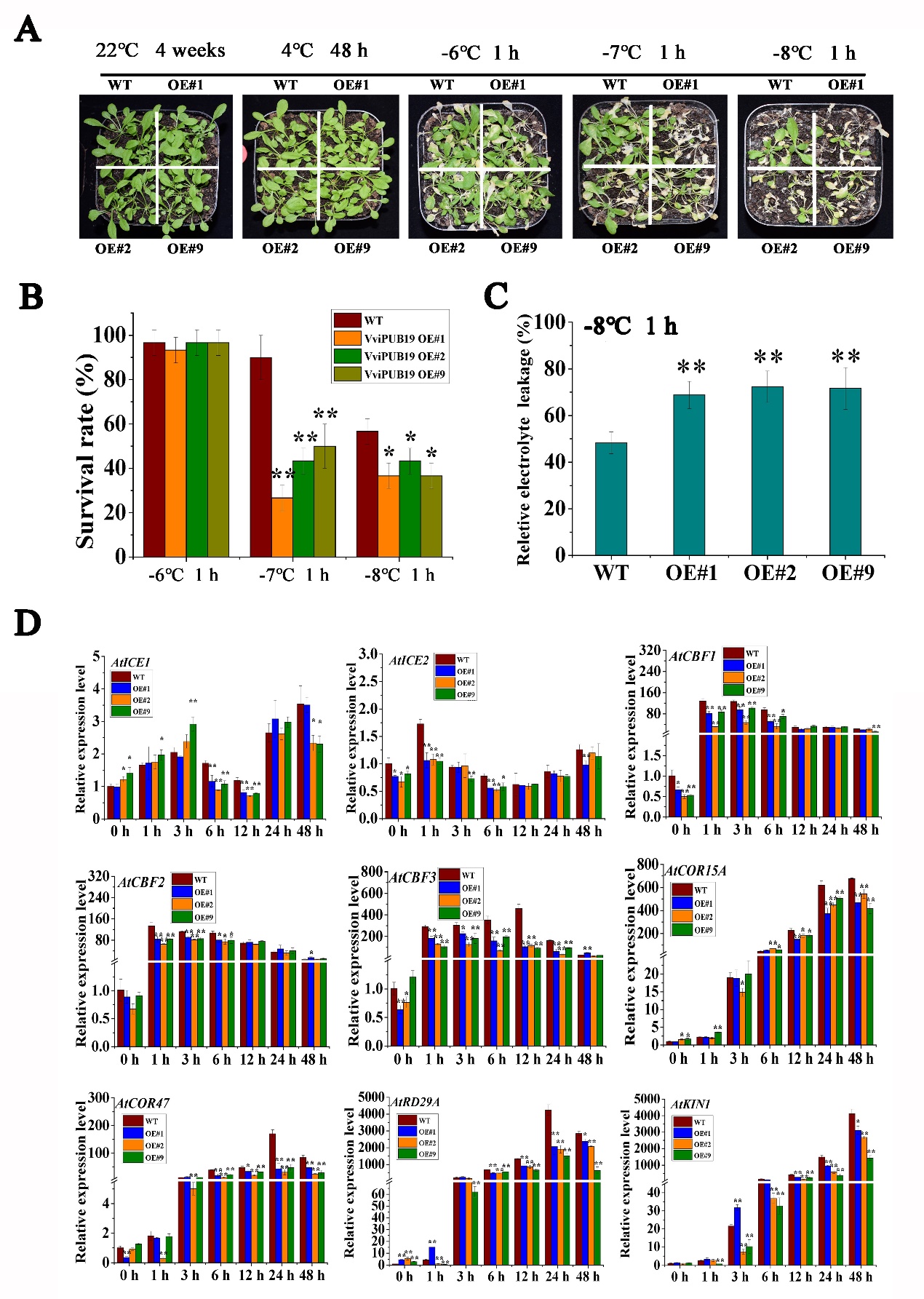


**Fig. S3.** **Analysis of cold resistance in *Arabidopsis thaliana* overexpressing *VviPUB19*. A,** **B and C.** Analysis of phenotype (A), survival (B) and relative electrolyte leakage (C) of 4-week-old wild-type (WT) and three *VviPUB19*-overexpressing Arabidopsis lines (OE#1, OE#2 and OE#9) under freezing treatment after acclimation at 4°C for 48 h; **D.** expression analysis of Cold -related gene in WT and *VviPUB19*-overexpressing Arabidopsis lines under 4°C treatment. Values shown are mean ± SD from three biological replicates. Significant differences are indicated with an asterisk (*t*-test, **P* < 0.05 or ***P* < 0.01).


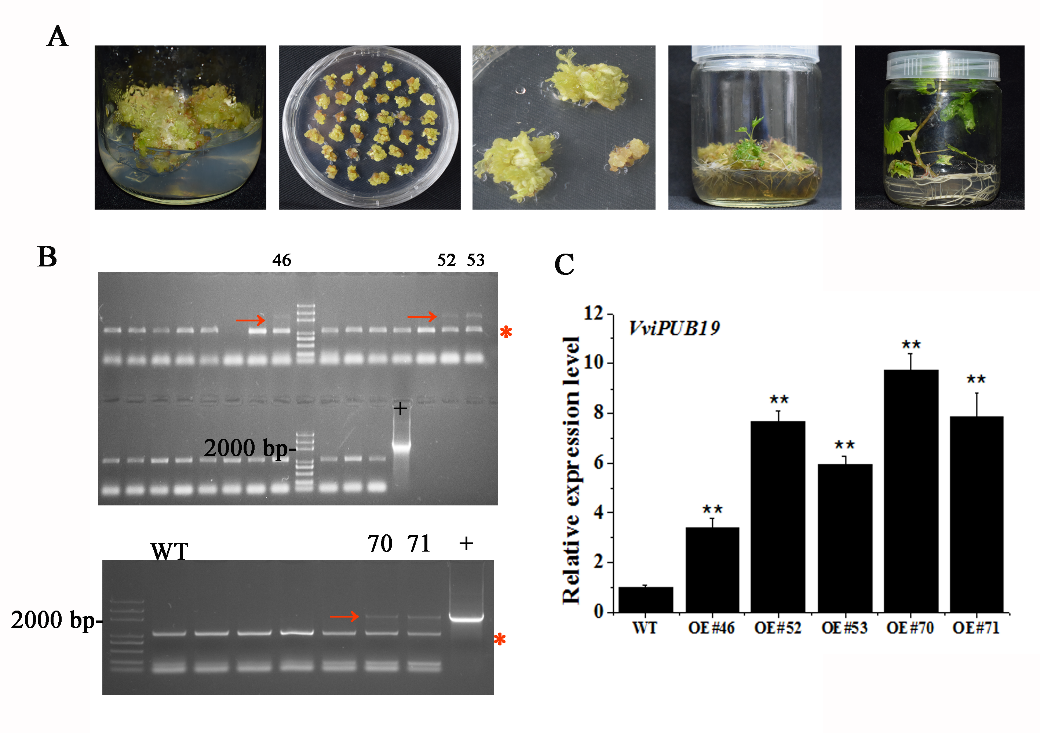


**Fig. S4.** **Genetic transformation and detection of *VviPUB19* overexpressing grape. A.** The genetic transformation process of grape organogenesis pathway; **B.** *VviPUB19* transgenic grape DNA detection, * indicates non-specific band; **C.** *VviPUB19* transgenic grape RNA detection. Values shown are mean ± SD from three biological replicates. Significant differences are indicated with an asterisk (*t*-test, **P* < 0.05 or ***P* < 0.01).


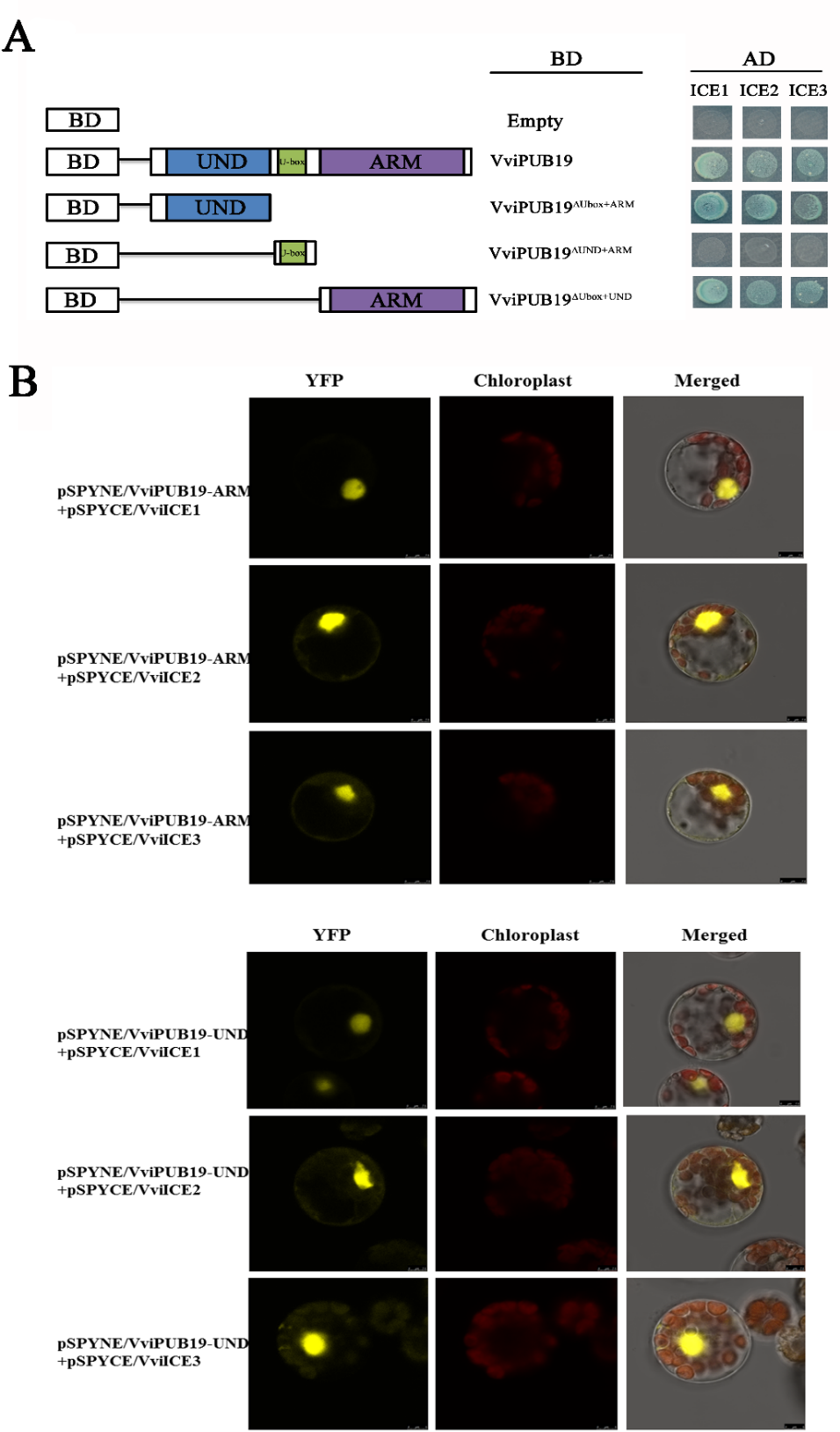


**Fig. S5. Interaction analysis of the conserved domain of VviPUB19 with VviICE1, 2 and 3.** A. the conserved domain of VviPUB19 interacts with VviICE1, 2 and 3 in the yeast two hybrid assay. A different prey vector and bait vector were used to co-transform Y2H yeast cells. Then single colonies were spotted on SD/-Ade/-His/-Leu/-Trp/X-α-Gal+200 ng/mL AbA medium to verify the interaction between proteins. B. BiFC assays verify the interaction between the conserved domain of VviPUB19 and VviICE1, 2 and 3. The plasmids were co-transformed into protoplasts mediated by PEG, and YFP fluorescence was observed after culture in darkness for 20 hours.


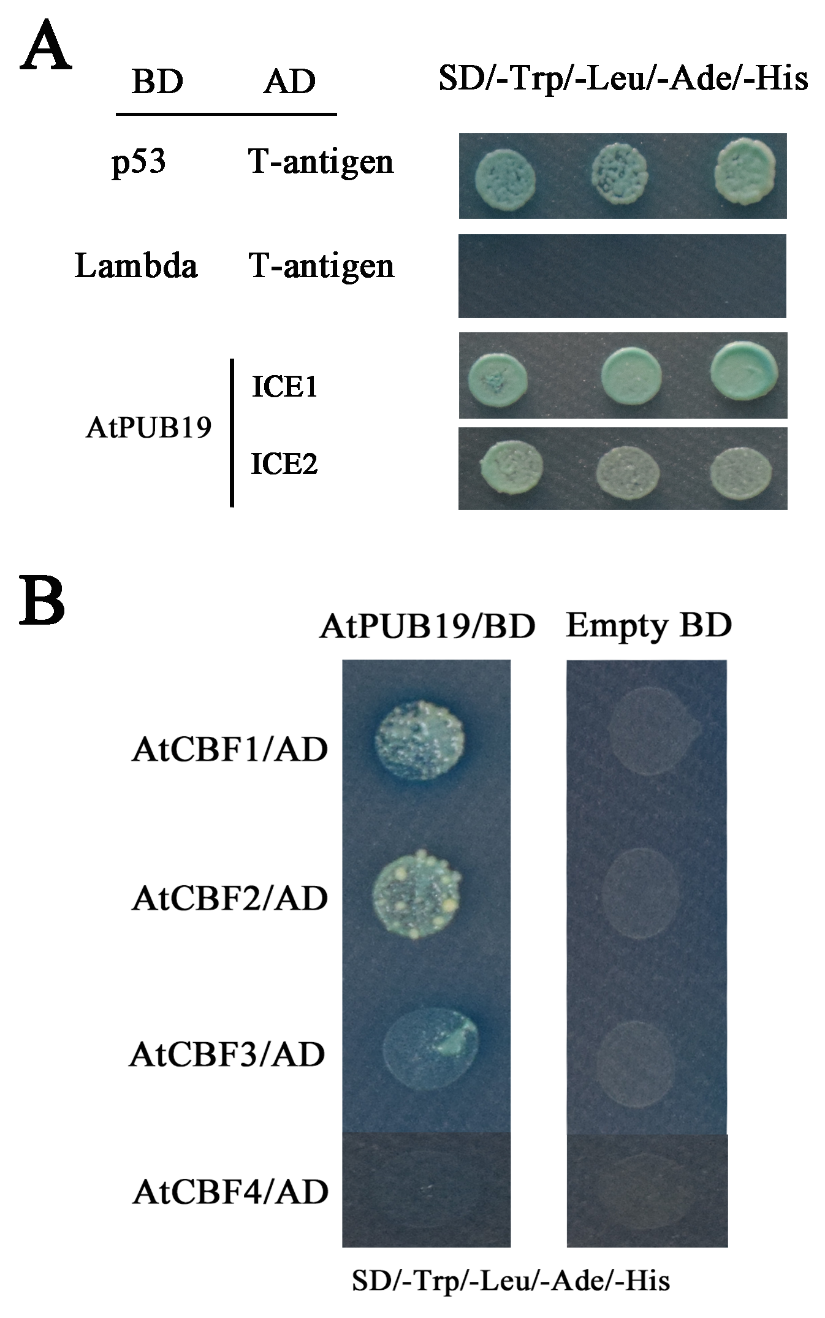


**Fig. S6.** **Interaction analysis of AtPUB19 with AtICEs and AtCBFs.** A. AtPUB19 interacts with AtICE1 and AtICE2. B. AtPUB19 interacts with AtCBF1 and AtCBF2. A different prey vector and bait vector were used to co-transform Y2H yeast cells. Then single colonies were spotted on SD/-Ade/-His/-Leu/-Trp/X-α-Gal+200 ng/mL AbA medium to verify the interaction between proteins.


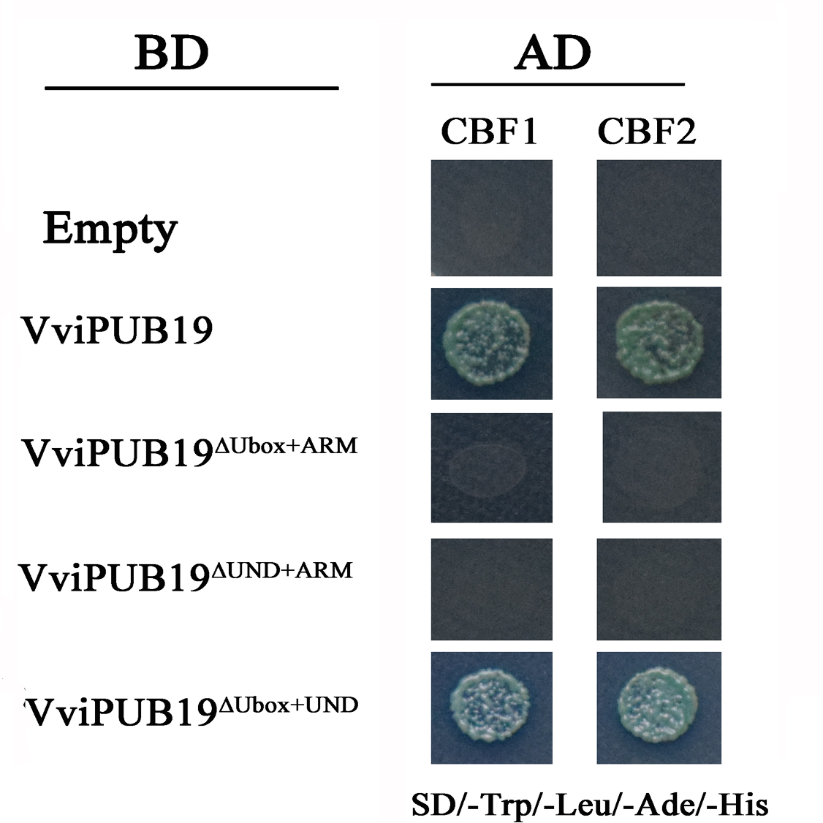


**Fig. S7.** **Interaction analysis of VviPUB19 with VviCBF1 and VviCBF2**. A different prey vector and bait vector were used to co-transform Y2H yeast cells. Then single colonies were spotted on SD/-Ade/-His/-Leu/-Trp/X-α-Gal+200 ng/mL AbA medium to verify the interaction between proteins.


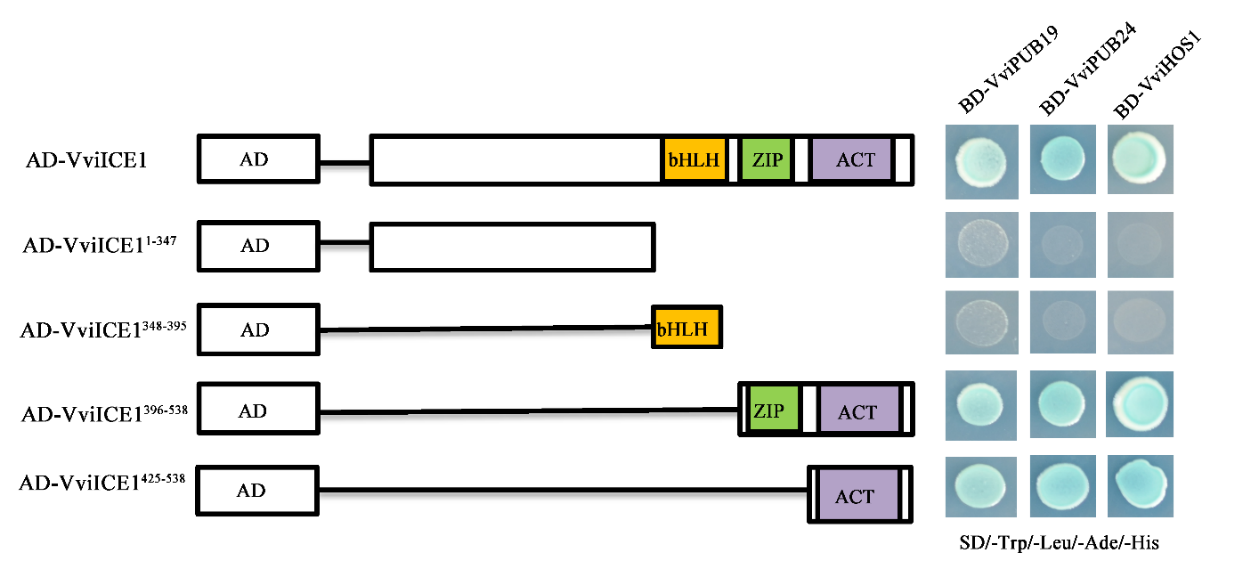


**Fig. S8.** **Interaction analysis of VviPUB19 with the domain of VviICE1.** A different prey vector and bait vector were used to co-transform Y2H yeast cells. Then single colonies were spotted on SD/-Ade/-His/-Leu/-Trp/X-α-Gal+200 ng/mL AbA medium to verify the interaction between proteins.


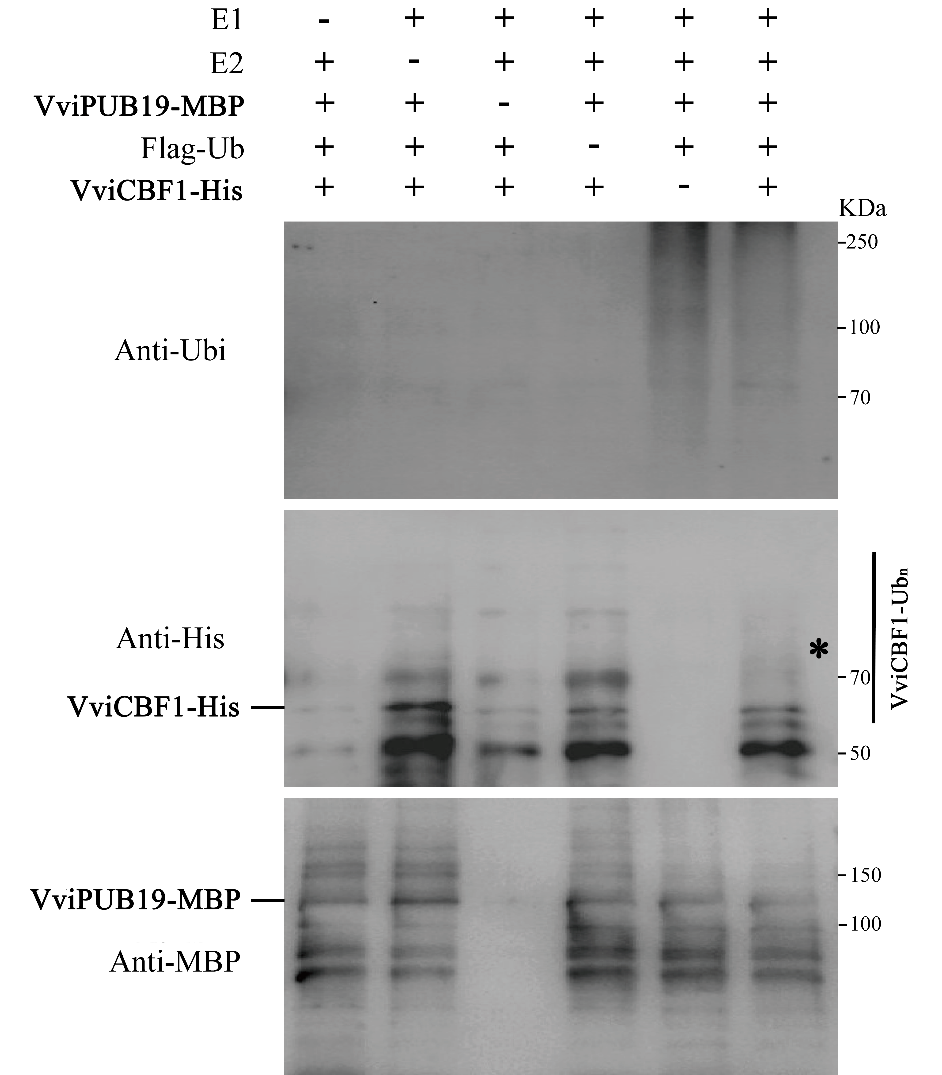


**Fig. S9.** ***In vitro* ubiquitination assay between VviPUB19 and VviCBF1.** In the presence of El, E2 and Ubiquitin proteins, the purified recombinant protein of VviCBF1-His was ubiquitinated by VviPUB19-MBP. The proteins were detected using anti-MBP, anti-His and anti-Ubiquitin antibodies. The asterisk represents the VviCBF1 ubiquitination polymerization.
