# Supplementary material for: The ubiquitin ligase VviPUB19 negatively regulates grape cold tolerance by affecting the stability of ICEs and CBFs
Source: Hortic Res. 2024 Oct 23;12(2):uhae297. doi: 10.1093/hr/uhae297 (PMC11822393; doi:10.1093/hr/uhae297)
Supplement: Web_Material_uhae297 [file web_material_uhae297.zip › Supplementary Tables.docx]

**Table S1.** The gene IDs and primers for the RT-qPCR analysis

| Name of primers | Primer sequence 5’-3’ | Gene IDs |
| --- | --- | --- |
| RT-VviActin-F | CCATCCTTCGTCTTGACCTTGCTG | AY680701 |
| RT-VviActin-R | AGTGGTGAACATGTAACCCCTCTC |  |
| RT-AtActin-F | CTTGCACCAAGCAGCATGAA | [AT3G18780](https://www.arabidopsis.org/servlets/TairObject?type=locus&name=AT3G18780) |
| RT-AtActin-R | CCGATCCAGACACTGTACTTCCTT |  |
| RT-VviPUB19-F | CCTAGGAACGGGAGCTTTACATCTC | [XM_002264601](https://www.ncbi.nlm.nih.gov/nucleotide/XM_002264601.5?report=genbank&log$=nucltop&blast_rank=2&RID=F486BCR3013) |
| RT-VviPUB19-R | TTGATGCACAGAGCCAACAACAGTG |  |
| RT-AtPUB19-F | GCGACGAAACAGCATTGTGTTGCTC | AT1G60190 |
| RT-AtPUB19-R | CGGTTTTCCTCTCCTGAAACTCGTG |  |
| RT-VviCBF1-F | GCAGAAGACATACAAGTAGCAGCGC | KF582113 |
| RT-VviCBF1-R | AGTTTCTAGTACCTTCTTCGAGTCTGC |  |
| RT-VviCBF2-F | GTCAGCAGGGTCGAGGAAGATAC | AY390376 |
| RT-VviCBF2-R | AACCATCTCCGGCTTTCTCCCAG |  |
| RT-VviCBF3-F | GCCATGTCATCTTCAGCTAGGGAC | KF758762 |
| RT-VviCBF3-R | CATCAGGCAGTGACATGTGAAATATGAGA |  |
| RT-VviCOR27A-F | GAGGGTTGTGGGAGTGCG | XM_003631622 |
| RT-VviCOR27A-R | AACTGTGCTTCTCTGTCCAT |  |
| RT-VviCOR27B-F | TCACTGTCATTTGCGCCAACGAGATT | XM_010666267 |
| RT-VviCOR27B-R | CAGTGACAGTATGTCTGTGAGAAGGA |  |
| RT-VviKIN2-F | AGATGTTTCCTTCCAAGCTGGTGAG | XM_002270462 |
| RT-VviKIN2-R | CAGCCTCTTGGCATGATTCCTTTGC |  |
| RT-VviLEA2-F | AGTGCATGACGCTGATGCCCTC | XM_034825923 |
| RT-VviLEA2-R | CTTCGACCGGCTCAGCGGC |  |
| RT-VviSTS5-F | TGAGTATGGTAACATGTCTAGTGCATG | XM_002278318 |
| RT-VviSTS5-R | GCAGCACAACGGTCTCAATGGTC |  |
| RT-AtCBF1-F | ACTTCGCTGACTCGGCTTGG | AT4G25490 |
| RT-AtCBF1-R | ACGCACCTTCGCTCTGTTCC |  |
| RT-AtCBF2-F | GGAATCAACCTGTGCCAAGGAA | [AT4G25470](https://bar.utoronto.ca/thalemine/portal.do?externalids=AT4G25470) |
| RT-AtCBF2-R | CCAACATCGCCTCTTCATCCATAT |  |
| RT-AtCBF3-F | TTCCTCAGGCGGTGATTATATTCC | [AT4G25480](https://www.arabidopsis.org/servlets/TairObject?type=locus&name=AT4G25480) |
| RT-AtCBF3-R | CTCCGACGAACTCCTCTGTATATTG |  |
| RT-AtCOR15A-F | AGTCGTTGATCTACGCCGCTAA | AT2G42540 |
| RT-AtCOR15A-R | TCTCACCATCTGCTAATGCCTCTT |  |
| RT-AtCOR47-F | CCAGGACACCACGACAAGACA | AT1G20440 |
| RT-AtCOR47-R | TCCACCACACTCTCCGACACT |  |
| RT-AtRD29A-F | GTTACTGATCCCACCAAAGAAGA | [AT5G52310](https://www.arabidopsis.org/servlets/TairObject?type=locus&name=AT5G52310) |
| RT-AtRD29A-R | GGAGACTCATCAGTCACTTCCA |  |
| RT-AtKIN1-F | CATCTCTTCTCATCATCACTAAC | [AT5G15960](https://www.arabidopsis.org/servlets/TairObject?type=locus&name=AT5G15960) |
| RT-AtKIN1-R | AACATTGCTCTTCTCCTCAG |  |

**Table S2.** Primers for vector construction

| Name of primers | Primer sequence 5’-3’ |
| --- | --- |
| P_VviPUB19_-GUS-F-Pst I | CCAAGCTTGGCTGCAGCCATGAAGCTCTACTTACGCACAAGAAT |
| P_VviPUB19_-GUS-R-EcoR I | ACTCCTCTTAGAATTCGGGAAAACAATTGAAAAAGGCTTTTCAAATT |
| VviPUB19-GFP-F-Kpn I | GGGGACGAGCTCGGTACCATGACTCACAAAATTCACGCCTCC |
| VviPUB19-GFP-R-Sal I | GCTCACCATGGTGTCGACGCCATACGTCAACATATCTTTCTTCTGG |
| VviPUB19-BD-F-EcoR I | ATGGCCATGGAGGCCGAATTCATGACTCACAAAATTCACGCCTCC |
| VviPUB19-BD-R-BamH I | CCGCTGCAGGTCGACGGATCCGCCATACGTCAACATATCTTTCTTCTGG |
| VviPUB19^∆U-box+ARM^-BD-F-EcoR I | ATGGCCATGGAGGCCGAATTCATGACTCACAAAATTCACGCCTCC |
| VviPUB19^∆U-box+ARM^-BD-R-BamH I | CCGCTGCAGGTCGACGGATCCGATCGGGGTTAAGGCAATTAAGCA |
| VviPUB19^∆UND+ARM^-BD-F-EcoR I | ATGGCCATGGAGGCCGAATTCGATTTTCGATGTCCTATCACTCTTG |
| VvPUB19^∆UND+ARM^-BD-R-BamH I | CCGCTGCAGGTCGACGGATCCGGTACTGCTGGATAAGCCTCTGGA |
| VviPUB19^∆UND+U-box^-BD-F-EcoR I | ATGGCCATGGAGGCCGAATTCTGTTCTGCTAATGGTATTCCAATTT |
| VviPUB19^∆UND+U-box^-BD-R-BamH I | CCGCTGCAGGTCGACGGATCCGCCATACGTCAACATATCTTTCTTCTGG |
| VviICE1-AD-F-EcoR I  VviICE1-AD-R-BamH I | GCCATGGAGGCCAGTGAATTCATGTTACCCAGGTCGAACGACG  CAGCTCGAGCTCGATGGATCCGCTACATCACACCATGGAAGCCG |
| VviICE2-AD-F-EcoR I  VviICE2-AD-R-BamH I | GCCATGGAGGCCAGTGAATTCATGCTGTCCAGAGTGAACGGC  CAGCTCGAGCTCGATGGATCCGCTACAGCATACCGTGGAAGCCT |
| VviICE3-AD-F-EcoR I  VviICE3-AD-R-BamH I | GCCATGGAGGCCAGTGAATTCATGTTGTCGGGGGTGAACGG  CAGCTCGAGCTCGATGGATCCGCTACATCATGCCATGGAAACCTGCT |
| VviCBF1-AD-F-EcoR I  VviCBF1-AD-R- BamH I | GCCATGGAGGCCAGTGAATTCATGGACTCGGACCATGAAGAGTT  CAGCTCGAGCTCGATGGATCCGCTAATCATCATTCCACAAAGACAAGTC |
| VviCBF2-AD-F-EcoR I  VviCBF2-AD-R- BamH I | GCCATGGAGGCCAGTGAATTCATGGACTTGGACCGTGAGTCTT  CAGCTCGAGCTCGATGGATCCGTTAAGATAGGAAATCATGATTCCACAAAGA |
| VviCBF3-AD-F-EcoR I  VviCBF3-AD-R- BamH I | GCCATGGAGGCCAGTGAATTCATGGAATCGGAGCGTGATCAGT  CAGCTCGAGCTCGATGGATCCGTTAATCATCATTCCACAAAGACAAGTCC |
| VviCBF4-AD-F-EcoR I | GCCATGGAGGCCAGTGAATTCATGAATACTACTTCTCCACCA |
| VviCBF4-AD-R-BamH I | CAGCTCGAGCTCGATGGATCCGTTATATTTTCCATTTTAAAAAGGAGA |
| VviPUB19-NE-F-BamH I | GCCTACTAGTGGATCCATGACTCACAAAATTCACGCCTC |
| VviPUB19-NE-R-Sal I | TACCCTCGAGGTCGACCCATACGTCAACATATCTTTCTTCTG |
| VviPUB19^∆U-box+ARM^-NE-F-BamH I | GCCTACTAGTGGATCCATGACTCACAAAATTCACGCCTC |
| VviPUB19^∆U-box+ARM^-NE-R-Sal I | TACCCTCGAGGTCGACATCGGGGTTAAGGCAATTAAGCA |
| VviPUB19^∆UND+U-box^-NE-F-BamH I | GCCTACTAGTGGATCCTGTTCTGCTAATGGTATTCCAATTT |
| VviPUB19^∆UND+U-box^-NE-R-Sal I | TACCCTCGAGGTCGACCCATACGTCAACATATCTTTCTTCTG |
| VviICE1-CE-F-Xba I | AACACGGGGGACTCTAGAGATGTTACCCAGGTCGAACGACG |
| VviICE1-CE-R-Kpn I | GTATGGGTACATGGTACCCATCACACCATGGAAGCCG |
| VviICE2-CE-F-Xba I | AACACGGGGGACTCTAGAGATGCTGTCCAGAGTGAACGGC |
| VviICE2-CE-R-Kpn I | GTATGGGTACATGGTACCCAGCATACCGTGGAAGCCT |
| VviICE3-CE-F-Xba I | AACACGGGGGACTCTAGAGATGTTGTCGGGGGTGAACGG |
| VviICE3-CE-R-Kpn I | GTATGGGTACATGGTACCCATCATGCCATGGAAACCTGCT |
| VviCBF1-CE-F-Xba I | AACACGGGGGACTCTAGAGATGGACTCGGACCATGAAGAGTT |
| VviCBF1-CE-R-Kpn I | GTATGGGTACATGGTACCATCATCATTCCACAAAGACAAGTC |
| VviCBF2-CE-F-Xba I | AACACGGGGGACTCTAGAGATGGACTTGGACCGTGAGTCTT |
| VviCBF2-CE-R-Kpn I | GTATGGGTACATGGTACCAGATAGGAAATCATGATTCCACAAAGA |
| VviICE1-myc-F-BamH I | GTATCTAGAACTAGTGGATCCATGTTACCCAGGTCGAACGACG |
| VviICE1-myc-R-Sal I | GGGCCCCCCCTCGAGGTCGACCTACATCACACCATGGAAGCCG |
| VviCBF1-myc-F-BamH I | GTATCTAGAACTAGTGGATCCATGGACTCGGACCATGAAGAGTT |
| VviCBF1-myc-R-Sal I | GGGCCCCCCCTCGAGGTCGACCTAATCATCATTCCACAAAGACAAGTC |
| VviPUB19-MBP-F-BamH I | GCGGCCGCGATATCGTCGACGGATCCATGACTCACAAAATTCACGCCTCCC |
| VviPUB19-MBP-R-EcoR I | CTTATTTAATTACCTGCAGGGAATTCCCATACGTCAACATATCTTTCTTCTGGAA |
| VviCBF1-His-F-Kpn I | AGGCATATGGAGCTCGGTACCATGGACTTGGACCGTGAGTC |
| VviCBF1-His-R-BamH I | GACAAGCTTGAATTCGGATCCATCATGATTCCACAAAGACAAGTC |
| P_VviPUB19-MYC_-pAbAi-F-Kpn I | TTCGAGCTCGGTACCAATGGAATAAGATATTGCAAGT |
| P_VviPUB19-MYC_-pAbAi-R-Sal I | ATGCCTCGAGGTCGACCCTCTAATTTAAAAAAAGTTTTGG |
| P_VviPUB19-MYC_-LUC-F-Sal I | GGGCCCCCCCTCGAGGTCGACCCATGAAGCTCTACTTACGCACAAGAA |
| P_VviPUB19-MYC_-LUC-R-BamH I | CGCTCTAGAACTAGTGGATCCGGGAAAACAATTGAAAAAGGCTTTTCA |
| VviICE1-SK-F-EcoR I | TCCCCCGGGCTGCAGGAATTCATGTTACCCAGGTCGAACGACG |
| VviICE1-SK-R-Kpn I | TGATTTCAGCGAATTGGTACCCATCACACCATGGAAGCCG |
| VviICE2 -SK-F-EcoR I | TCCCCCGGGCTGCAGGAATTCATGCTGTCCAGAGTGAACGGC |
| VviICE2-SK-R-Kpn I | TGATTTCAGCGAATTGGTACCCAGCATACCGTGGAAGCCT |
| VviICE3 -SK-F-EcoR I | TCCCCCGGGCTGCAGGAATTCATGTTGTCGGGGGTGAACGG |
| VviICE3-SK-R-Kpn I | TGATTTCAGCGAATTGGTACCCATCATGCCATGGAAACCTGCT |
